# Autoantibodies against Muscarinic Type 3 Receptor in Sjögren's Syndrome Inhibit Aquaporin 5 Trafficking

**DOI:** 10.1371/journal.pone.0053113

**Published:** 2013-01-30

**Authors:** Byung Ha Lee, Adrienne E. Gauna, Geidys Perez, Yun-jong Park, Kaleb M. Pauley, Toshihisa Kawai, Seunghee Cha

**Affiliations:** 1 Department of Oral and Maxillofacial Diagnostic Sciences, University of Florida College of Dentistry, Gainesville, Florida, United States of America; 2 Department of Science and Mathematics, Cedarville University College of Arts and Sciences, Cedarville, Ohio, United States of America; 3 Department of Immunology, The Forsyth Institute, Cambridge, Massachusetts, United States of America; University Paris Sud, France

## Abstract

Sjögren's syndrome (SjS) is a chronic autoimmune disease that mainly targets the salivary and lacrimal glands. It has been controversial whether anti-muscarinic type 3 receptor (α-M3R) autoantibodies in patients with SjS inhibit intracellular trafficking of aquaporin-5 (AQP5), water transport protein, leading to secretory dysfunction. To address this issue, GFP-tagged human AQP5 was overexpressed in human salivary gland cells (HSG-hAQP5) and monitored AQP5 trafficking to the plasma membrane following carbachol (CCh, M3R agonist) stimulation. AQP5 trafficking was indeed mediated by M3R stimulation, shown in partial blockage of trafficking by M3R-antagonist 4-DAMP. HSG-hAQP5 pre-incubated with SjS plasma for 24 hours significantly reduced AQP5 trafficking with CCh, compared with HSG-hAQP5 pre-incubated with healthy control (HC) plasma. This inhibition was confirmed by monoclonal α-M3R antibody and pre-absorbed plasma. Interestingly, HSG-hAQP5 pre-incubated with SjS plasma showed no change in cell volume, compared to the cells incubated with HC plasma showing shrinkage by twenty percent after CCh-stimulation. Our findings clearly indicate that binding of anti-M3R autoantibodies to the receptor, which was verified by immunoprecipitation, suppresses AQP5 trafficking to the membrane and contribute to impaired fluid secretion in SjS. Our current study urges further investigations of clinical associations between SjS symptoms, such as degree of secretory dysfunction, cognitive impairment, and/or bladder irritation, and different profiles (titers, isotypes, and/or specificity) of anti-M3R autoantibodies in individuals with SjS.

## Introduction

Sjögren's syndrome (SjS), a systemic autoimmune disease primarily targeting the salivary and lacrimal glands, results in severe dry mouth and dry eyes [1], [2]. Despite endeavors to define the environmental, genetic, physiological and immunological causes for SjS onset and progression, underlying etiologies remain poorly understood. Possible mechanisms for dry mouth and dry eyes include epithelial cell apoptosis by autoreactive T- or B-lymphocytes infiltrating the glands and pro-inflammatory cytokines [3], [4]. Another important mechanism underlying secretory dysfunction in SjS involves inhibitory roles of autoantibodies to the acetylcholine muscarinic type 3 receptor (M3R), which is critical for fluid secretion from acinar cells. It has been reported that autoantibodies against M3R suppress secretion by functioning as an antagonist for the receptor [5], [6], [7], [8]. In addition, studies have demonstrated acute inhibition of parasympathetic neurotransmission in bladder smooth muscle in the presence of SjS anti-M3R autoantibodies [9], [10].

M3R belongs to the subfamily of G protein coupled receptors (GPCR), which includes subtypes of M1R-M5R. M3R coupled to G_q/11_ and can initiate the phosphatidylinositol triphosphate cascade through the binding of muscarinic agonists such as acetylcholine or carbachol, and thus mediate Ca^2+^ release from intracellular calcium storage [11]. This elevated intracellular concentration of Ca^2+^ was shown to induce saliva secretion from rat parotid acinar cells [12], [13]. One of the main water transport proteins involving saliva secretion in response to increased concentration of Ca^2+^ is aquaporin 5 (AQP5) [13], [14]. It has been shown that rat AQP5 expressed in human salivary gland (HSG) cells translocate to the plasma membrane in response to increased intracellular Ca^2+^concentration [15].

Aquaporin proteins are widely distributed throughout the organs, playing various roles in the body as well as in salivation. Among the 13 known aquaporin proteins, AQP5 is detected in lungs, cornea, salivary, and lacrimal glands [16]. In the salivary glands, AQP5 is localized at the apical membrane of serous-type acinar cells [17]. AQP5 knockout studies in mice confirmed the critical role of AQP5 in water secretion in the salivary gland acinar cells [18], [19]. However, whether abnormal trafficking of AQP5 contributes to the loss of secretory function in SjS as a result of antagonist effects of anti-M3R autoantibodies on the receptor is still largely controversial. Studies indicate that different AQP5 staining methods on the salivary or lacrimal glands of SjS patients have produced inconsistent results; abnormal distribution/selective defect for AQP5 trafficking to apical membrane vs. no difference in the distribution and density of AQP5 in patients with primary SjS [20], [21], [22]. Studies with SjS mouse models have also shown conflicting observations with respect to AQP5 distribution in the salivary glands [23], [24].

Therefore, we investigated whether AQP5 trafficking is altered in SjS due to the presence of anti-M3R autoantibodies by monitoring GFP-tagged human AQP5 trafficking in cells pre-incubated with SjS plasma or sera under a confocal imaging system. We hypothesized that anti-M3R autoantibodies inhibit AQP5 trafficking to the apical membrane, thus contributing to secretory dysfunction in SjS.

## Materials and Methods

### HSG cell culture and transfection

Human submandibular gland (HSG) cell line, originally from NIDCR, was provided by Dr. Joseph Katz in Oral Medicine at the College of Dentistry [25]. The cells were maintained in Dulbecco's modified Eagle's medium supplemented with 10% fetal calf serum, 100 units/ml of penicillin, and 100 μg/ml of streptomycin (Life Technologies) at 37°C. For transfection, 50,000 HSG cells were seeded in each well of 8-chamber-well slides (BD bioscience) and cultured in growth media overnight. The cells were transfected with Lipofectamine™ 2000 (Invitrogen), according to manufacturer's protocol, with rhAQP5 expression vector (0.5 mg) or control GFP expression vector (0.5 mg) diluted in Opti-MEM serum-free medium (Invitrogen). Transfected HSG cells were incubated for 48 hours for the maximum transfection efficiency. To block M1R or M3R on HSG cells, pirenzepine dihydrochloride (pirenzepine, M1R antagonist, Sigma-Aldrich) or 4-diphenyl-acetoxy-N-methyl-piperidine (4-DAMP, M3R antagonist, Ascent Scientific LLC) at a 10 µM concentration was treated for 10 minutes. For the M3R stimulation on HSG cells, 100 μM of carbamylcholine chloride (carbachol, M3R agonist, Sigma-Aldrich) was treated for 10 minutes.

### Construction of GFP-tagged human aquaporin 5 (rhAQP5)

Messenger RNA from HSG cells was used to clone hAQP5 by reverse transcription polymerase chain reaction (RT-PCR). RT-PCR was performed using primers as follows: (F) 5′-CTCGAGATGAAGAAGGAGG-3′ and (R) 5′-GAATTCTATAGCGGGTGGTCAG-3′. PCR conditions were 5 min at 95°C, (30 s at 95°C, 30 s at 57°C, and 30 s at 72°C) ×30 times, and 7 min at 72°C. Purified RT-PCR product was sequenced and compared with NCBI database using nucleotide BLAST and cloned into multiple cloning site of pAcGFP1-N1 GFP expression vector (Clontech).

### Patient and control subjects

Five plasma samples from female patients with primary SjS selected for positive anti-M3R IgG at a plasma titration of 1/500. The patients were selected based on the modified European-American diagnostic criteria [26]. Patients' demographic, clinical and laboratory characteristics are summarized in Table 1. Five gender-matched healthy donors with no history of autoimmune diseases were included as healthy controls. This study was approved by the University of Florida Institutional Review Board, and a written permission was obtained from all who participated in the study.

**Table 1:** Demographics and clinical data for patients.

Subject	Sex	Age (Years)	Diagnosis	Medications	SSA*	SSB*	Biopsy/focus score	Salivary flow (ml/min)
SjS-1	F	65	Primary SjS	Cevimeline	Neg*	Neg	Pos*, 7	0.293
SjS-9	F	77	Primary SjS	None	Pos	N/A*	N/A	0.03
SjS-15	F	65	Primary SjS	Prednisone; Hydroxychloroquine; Methotrexate	Pos	Neg	N/A	0.011
SjS-16	F	78	Primary SjS	Hydroxychloroquine	Pos	Pos	Pos, 5	0.042
SjS-26	F	32	Primary SjS	Hydroxychloroquine; Cyclosporin opthalmic	Pos	Neg	Pos, 3	0.06

* N/A, no data available; Pos, positive; Neg, Negative; SSA, anti-Lo antibodies; SSB, anti-La antibodies.

### Immunofluorescent staining

Cells were fixed with 4% paraformaldehyde for 15 minutes or with PBS in case of live cell staining. Primary and secondary antibodies were incubated for 90 min. and 30 min., respectively, at room temperature in 1% BSA in PBS. M3R protein was detected with rabbit anti-M3R antibodies (Santa Cruz Biotechnology) (1∶100 dilution) or mouse monoclonal anti-hM3R loop2 antibody (1∶100 dilution) as a primary antibody and Alexa Fluor 568-labeled goat anti-rabbit or mouse IgG (Molecular Probes) (1∶400 dilution) as a secondary antibody. For IgG detection, plasma samples (1∶500) from healthy controls or SjS patients for the fixed cells or purified IgG or pre-absorbed plasma for live cell staining were used as a primary antibody. Secondary antibody was Alexa Fluor 488-labeled goat anti-human IgG (1∶400 dilution) from Molecular Probes. Images were observed at a 200X or a 400X magnification using a Zeiss Axiovert 200M microscope equipped with a Zeiss AxioCam MRm camera and obtained with AxioVs40 software (Ver. 4.7.1.0, Zeiss). For IgG purification, five plasma samples in each group were pooled and purified using PureProteomeTM Protein G Magnetic Beads (Millopore).

### Western blotting and immunoprecipitation

For total cell lysates, cells were lysed in lysis buffer (50 mM Tris-HCl pH 7.5, 2 mM EDTA, 500 mM NaCl, and 1% NP-40) supplemented with protease inhibitors (complete Mini, Roche). To purify membrane fraction, Mem-PER Membrane Protein Extraction Kit (Pierce) was used according to manufacturer's instruction. Protein concentrations of cell lysates were determined by the Bradford protein assay, and 1 mg of each lysate preparation was separated by electrophoresis through 4–20% linear gradient Tris-HCl precast gels (Bio-Rad Laboratories). For immunoprecipitation, pooled HC or SjS plasma were mixed with and PureProteomeTM protein G magnetic Beads (Millipore) and washed five times according to manufacturer's instruction, then, mixed with M3R overexpressing HSG cell lysates and incubated overnight at 4°C. To overexpress M3R on HSG cells, 3x-hemagglutinin (3x-HA) tagged M3R expression vector (UMR cDNA Resource Center) was transfected into HSG cells. For western blotting, protein samples were resolved by SDS-PAGE then transferred to PVDF membranes (0.2 μm pore size, Bio-Rad Laboratories) using a Pierce Fast Semi-Dry Blotter (Thermo Scientific) and the membranes probed with either goat anti-human β-actin (Sigma-Aldrich) or goat anti-pan-cadherin (Santa Cruz Biotechnology) or rabbit anti-human AQP5 (Santa Cruz Biotechnology) or mouse anti-HA (Sigma-Aldrich) antibodies. The membranes were incubated with appropriate HRP-conjugated secondary antibodies. The signals were visualized using the ECL Advance Detection Kit (Thermo Scientific).

### Confocal Microscopy

HSG cells (2×10^5^ cells/well) were grown on Delta T culture dish (0.17 mm thick, Bioptechs) in a 6-well plate overnight. rhAQP5 expression vector (0.5 µg) was transfected into the cells in each well by Lipofectamine™ 2000 (Invitrogen). At 48 hour of post-transfection, HSG cells with hAQP5-GFP transfection were pre-incubated with plasma from five HC or five SjS patients for 24 hours and stimulated with CCh (100 µM). Live-cell images were acquired with a spinning disk confocal connected to a Leica DMIRB microscope with a 63X oil-immersion objective, using a cascade-cooled EMCCD camera (Photometrics, Tucson, AZ, USA), under the control of µManager open-source software (http://www.micro-manager.org/University of California San Francisco, CA, USA). Throughout the imaging process, the cells were maintained on a temperature-controlled stage at 37°C, and observed at 30 second intervals up to 30 min. with 1/250 second fixed exposure time. Representative images were constructed using the image analysis software ImageJ (http://rsb.info.nih.gov/ij/).

### Image Analyses

Representative confocal microscopy imagesshowing AQP5 trafficking in HSG cells were converted into ‘Fire’ 3D LUT (lookup tables) setting in ImageJ software for the signal quantification. GFP signal that was above the set fluorescent signal threshold (≥80, red or yellow colorin bar chart) was considered as positive for the analysis. Signal above the threshold along the membrane was calculated and presented as K, which is percentage of positive signal on the membrane, based on the following formula: positive signal on the membrane divided by positive signal on the total circumference length of membrane.

### Measurement of intracellular calcium release in HSG cells

HSG cells that were plated in 96 well plates one day before (20,000 cells/well) were pre-incubated with IgG fraction (10 μg/ml) from HC or SjS plasma, or monoclonal anti-M3R antibody (10 μg/ml) for 24 hours. Then, HSG cells were stained and incubated for calcium release assay using Fluo-4 NW Calcium Assay Kits (Molecular ProbesTM) according to manufacturer's instruction. Carbachol (CCh, 100 μM) or Ionomycin (positive control, 100 μM) was applied to HSG cells to stimulate calcium release in HSG cells. Relative intracellular calcium concentration [Ca^2+^] was measured as fluorescence signal within a minute using Spectramax M5 Microplate Reader (Molecular devices). The fluorescence detected using instrument settings appropriate for excitation at 494 nm and emission at 515 nm and analyzed by using softmax pro 4.8 software.

### Statistical Analyses

The data are expressed as the mean ± SEM. Statistical evaluation was determined by using t-test by the GraphPad Prism software (GraphPad Software). The p value less than 0.05 was considered statistically significant.

## Results

### Muscarinic receptor agonist, carbachol, induces rhAQP5 trafficking in human salivary gland (HSG) cells

**Figure 1:**
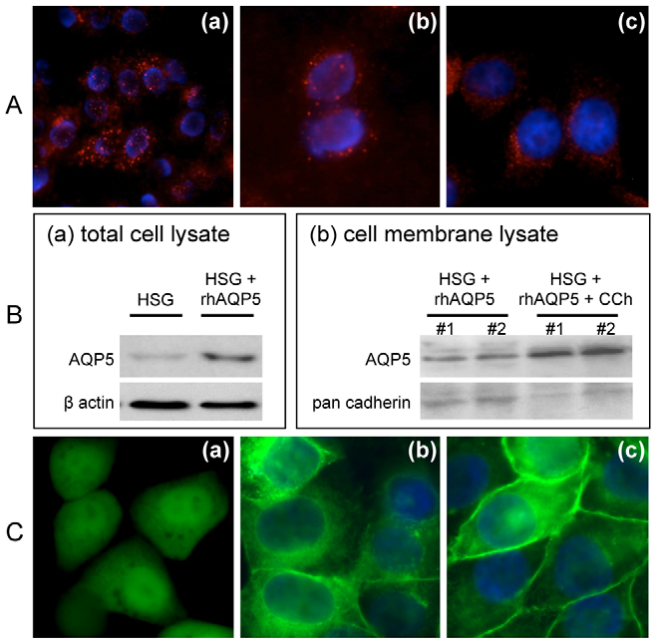
Increased membrane expression of GFP-tagged-recombinant human aquaporin 5 (**rhAQP5**) **following carbachol stimulation in HSG cells.** (A) M3R expression was detected in HSG cells (red color) using polyclonal (a, b) or monoclonal (c) antibody by immunocytochemistry. Stained cells were observed at ×200 (a) and ×400 (b, c) magnifications. (B) hAQP5 protein expressions were examined by western blotting in total HSG cell lysates purified from non- or rhAQP5 vector-transfected HSG cells (a) and in HSG cell membrane fractions purified from transfected cells with or without CCh treatment (b) in duplicates #1 and #2. (C) To confirm the hAQP5 trafficking in HSG cells, control GFP (a) or rhAQP5 (c) vector transfected HSG cells were treated with carbachol (CCh, 100 μM) (a and c) or no stimulation (b). Images were taken under microscope with a ×400 magnification using a Zeiss Axiovert 200M microscope and obtained with AxioVs40 software (Ver. 4.7.1.0, Zeiss).

The expression of M3R mRNA in human salivary gland (HSG) cells was confirmed in previous studies [27], [28]. We also screened all five muscarinic receptor expressions (M1R-M5R) in HSG cells and confirmed weak mRNA expression of M1R and major expression of M3R (Figure S1). To confirm the expression of M3R protein on HSG cells, we performed immunofluorescence staining on HSG cells by using a commercially available polyclonal antibody specific for M3R or monoclonal antibody against 2^nd^ extracellular loop of human M3R. Figure 1A shows the expression of M3R on the HSG cell membrane (red signal) detected by commercially available anti-M3R antibodies (a, b) or monoclonal antibody (c) at ×200 (a) or ×400 (b, c) magnifications. After confirming the presence of M3R on HSG cells, GFP-tagged recombinant human AQP5 expression vector (rhAQP5) was constructed and transfected into HSG cells (rhAQP5-HSG) to monitor AQP5 trafficking. Figure 1B-(a) shows expression of hAQP5 in total cell lysates from HSG or rhAQP5-HSG cells at 48-hour post transfection by western blotting. The expression was significantly increased after transfection in comparison with endogenous expression of AQP5 in non-transfected HSG cells. Next, we tested hAQP5 trafficking in rhAQP5-HSG cells following carbachol (CCh, an acetylcholine receptor agonist) stimulation. To compare the hAQP5 expression on the membrane with or without CCh treatment on rhAQP5-HSG cells, membrane fractions were isolated and hAQP5 protein expressions were detected by western blotting (Figure 1B-(b)). Increased expression of hAQP5 in the membrane of CCh treated rhAQP5-HSG cells confirmed translocation of hAQP5 from cytosol into membrane.

To visualize the hAQP5 translocation to HSG cell plasma membrane following CCh stimulation, HSG cells were seeded (50,000 cells/well) on an 8-well chamber slide and transfected with control GFP or rhAQP5 vector, cells were stimulated with or without CCh (100 μM) for 10 minutes. Then, HSG cells were fixed and observed under the microscope at a ×400 magnification. As shown in Figure 1C, control vector transfected HSG cells uniformly expressed GFP signal and showed no changes in GFP signal followed by CCh treatment (Figure 1C-(a)) and rhAQP5-HSG cells showed rhAQP5-GFP expression mainly in cytosolic region (Figures 1C-(b)). However, CCh induced rhAQP5-HSG cells showed hAQP5 trafficking to the membrane (Figure 1C-(c)), which indicated as enhanced green signals on the cell membrane. This confirmed that agonist CCh stimulation on muscarinic receptors (MRs) translocates hAQP5 to the plasma membrane of HSG cells (CCD captured real-time image shown in Figure S2).

### Pre-incubation of a M3R-specific antagonist, 4-DAMP, inhibited CCh-induced hAQP5 trafficking in rhAQP5-transfected HSG cells

**Figure 2:**
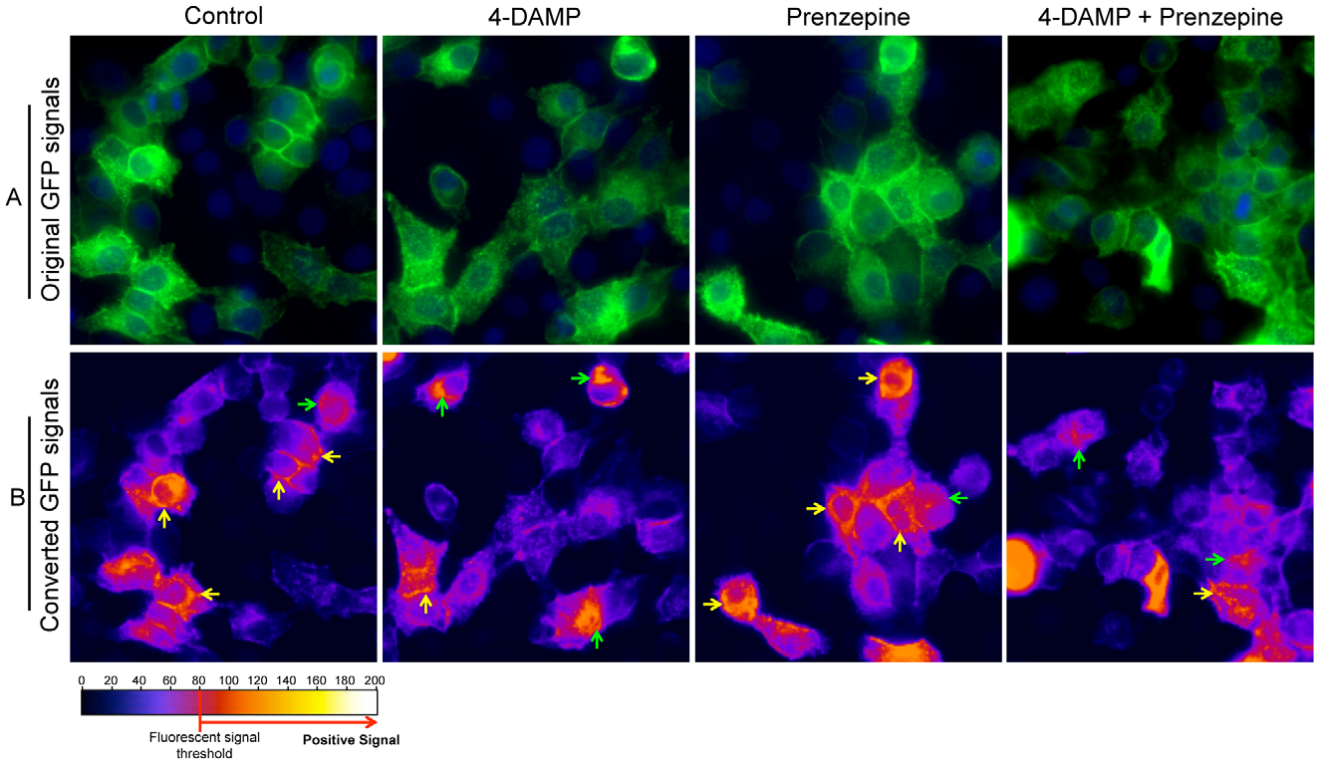
Inhibition of hAQP5 trafficking in rhAQP5 vector-transfected HSG cells in the presence of 4-DAMP. (A) rhAQP5 vector-transfected HSG cells were stimulated with CCh (100 μM) in the presence or absence of 4-DAMP (10 μM), prenzepine (10 μM), and mixture of 4-DAMP and prenzepine (M3R or M1R specific antagonist, respectively). (B) To monitor hAQP5 trafficking, original GFP pictures in (A) were converted into ‘Fire’ 3D LUT (lookup tables) setting in ImageJ software and only GFP signal above the fluorescent signal threshold (≥80, red or yellow color in converted picture), was captured in (B) as positive GFP signal. Yellow arrows indicate the positive GFP signal on the membrane and green arrows indicates signal in the cytoplasm in HSG cells. Cells were observed at ×200 magnification using a Zeiss Axiovert 200M microscope and images were obtained with AxioVs40 software (Ver. 4.7.1.0, Zeiss).

After confirmation of hAQP5 trafficking to HSG cell membrane following CCh treatment, we next sought to determine if hAQP5 trafficking would be inhibited or interfered by the blocking M3R via a M3R-specific antagonist. Among the muscarinic receptor antagonists, 4-diphenyl-acetoxy-N-methyl-piperidine (4-DAMP) is known as a M3R-specific antagonist for intracellular Ca^2+^ signaling cascade [9], [11]. Nagy et al. has confirmed blocking intracellular Ca^2+^ release by 4-DAMP against M3R in HSG cells [27]. To verify AQP5 trafficking is mediated by CCh-induced calcium release via M3R, rhAQP5 vector-transfected HSG cells were pre-treated with 4-DAMP (10 μM) or prenzepine, M1R specific antagonist, (10 μM), or 4-DAMP/prenzepine cocktail for 10 minutes. Then, cells were treated with CCh (100 μM) for 10 minutes and fixed with 4% of paraformaldehyde to examine the AQP5 trafficking. hAQP5 trafficking in rhAQP5-HSG cells after CCh stimulation in the presence or absence of M3R or M1R antagonist (4-DAMP or prenzepine, respectively) was shown in Figure 2A. To monitor or compare hAQP5 trafficking to the cell membrane, original images with GFP signal in Figure 2A were converted into ‘Fire’ 3D LUT (lookup tables) setting in ImageJ software and only GFP signal above the fluorescent signal threshold (≥80, red or yellow color in converted picture), was considered as a positive GFP signal. In Figure 2B, yellow arrows indicate the positive GFP signal on the membrane and green arrows indicate positive signals in the cytoplasm. Following CCh stimulation, 4-DAMP pre-incubation suppressed hAQP5 trafficking to HSG cell membrane compared to AQP5 trafficking in the control. The majority of HSG cells showed suppressed or inhibited hAQP5 trafficking to plasma membrane with a small fraction of cells exhibiting normal level of signal in the membrane. In contrast, cells pre-incubated with M1R antagonist prenzepine still exhibited strong signals in the membrane but when treated in combination with 4-DAMP, it reduced the signal in the membrane. These results strongly indicate that although other MR subtypes, such as M1R, are present on HSG cells, hAQP5 trafficking is mainly mediated through M3R stimulation with CCh.

### SjS IgG binds to M3R and other surface antigens on HSG cells

**Figure 3:**
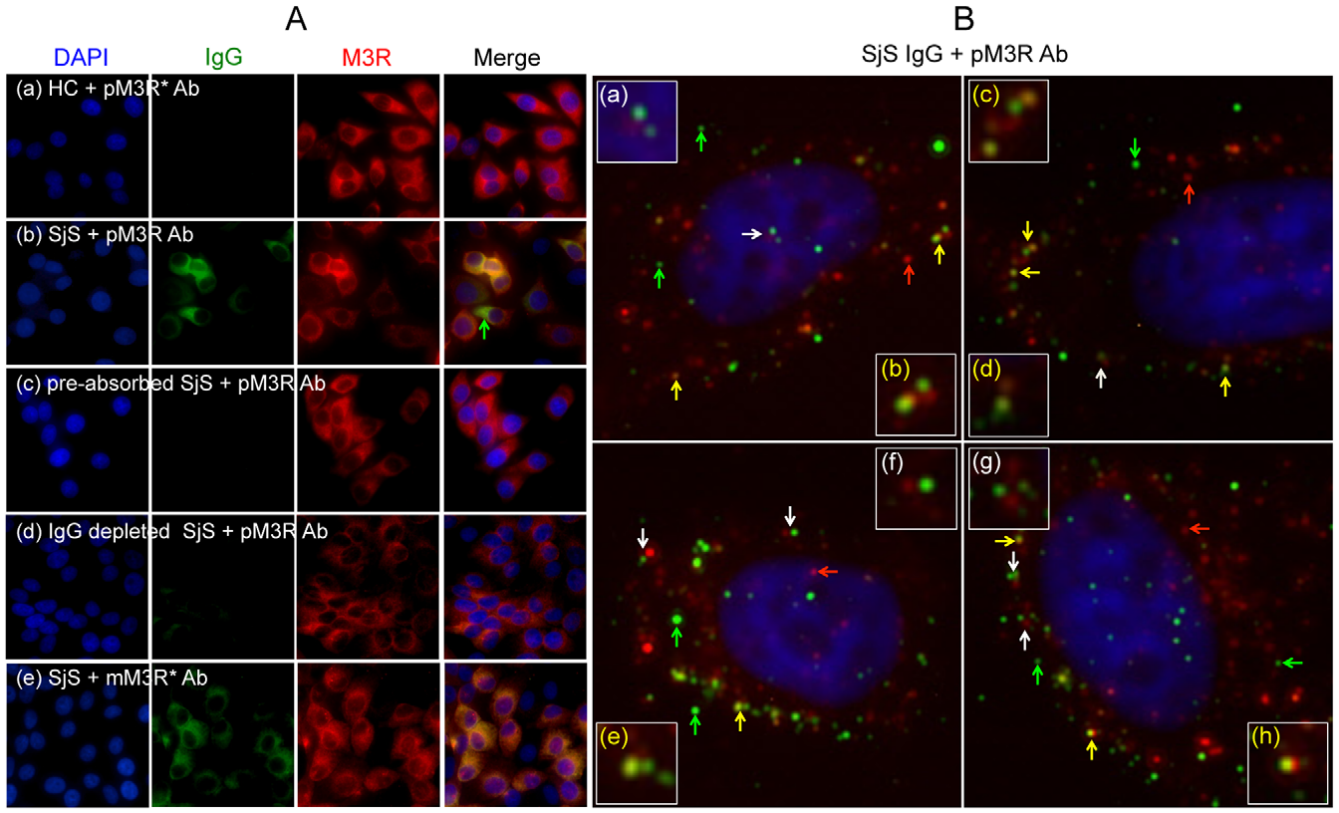
Presence of anti-M3R autoantibodies in SjS plasma detected by immunocytochemistry. (A) HSG cells were pre-incubated with (a) healthy control (HC) plasma, (b, e) Sjögren's syndrome (SjS) plasma, (c) pre-absorbed SjS plasma, or (d) IgG depleted SjS plasma for 24 hours. Endogenous expressions of M3R (red color) on HSG cells were detected by using poly- (a–d) or mono-(e) clonal antibody. Immunofluorescence staining of HSG cells following pre-incubation with SjS plasma indicated co-localization [yellow or orange color in merged picture (b) and (e)] of human IgG (green color) and M3R (red color) on HSG cells. This specific binding was confirmed by pre-absorbed (c), IgG depleted plasma (d), or monoclonal antibody specific for M3R (e). Stained cells were observed at a ×200 magnification. (pM3R* Ab: polyclonal anti-hM3R antibody; mM3R Ab: monoclonal anti-hM3R antibody). (B) HSG cells were incubated with purified IgG from five pooled-SjS plasma samples and polyclonal anti-M3R antibody followed by Alexa Fluor 488 and 568-labeled secondary antibody incubation, respectively. Yellow (b, c, d, e, and h) or white arrows (a, f, and g) in the image picture indicate co-localization or adjacent staining of IgG and M3R, respectively. poly α-M3R ab, polyclonal antibody; mono α-M3R ab, monoclonal antibody; Green arrows, IgG staining; red arrows, M3R staining; white arrows, M3R and IgG adjacent staining; yellow arrows, co-localization of IgG and M3R. Cells were observed at ×400 magnification using a Zeiss Axiovert 200M microscope and images were obtained with AxioVs40 software (Ver. 4.7.1.0, Zeiss).

To investigate the roles of anti-M3R autoantibodies in regulating AQP5 trafficking, five healthy control (HC) or five SjS patient plasma samples were selected following screening (Table 1). All five SjS plasma samples obtained from female patients with primary SjS were positive for IgG that binds to HSG cells at a plasma titration of 1/500. Only HSG cells pre-incubated with SjS plasma showed positive IgG staining (Figure 3A-(b) and (e)) while HSG cells incubated with HC plasma were negative (Figure 3A-(a)). Furthermore, HSG cells pre-incubated with SjS plasma showed co-localization of IgG and M3R as indicated in Figure 3A (yellow in the merged image). However, when this plasma sample was pre-absorbed for 24 hours with HSG cells, IgG binding was no longer detected, indicating that SjS plasma contains anti-M3R autoantibodies. In addition, cells pre-incubated with IgG-depleted plasma, which a byproduct of IgG purification showed negative IgG staining on the surface as expected. Interestingly, HSG cells pre-incubated with a SjS plasma sample showed green color in a merged image, indicating presence of IgG specific to other unidentified antigens besides M3R in SjS plasma (green arrows in Figure 3A). This green signal was not detected, as expected, in HSG cells pre-incubated with monoclonal antibody since the antibody was raised specifically for M3R.

To further verify the presence of anti-M3R autoantibodies in SjS plasma, IgG was purified from five SjS plasma samples and used as primary antibody in combination with commercially available anti-M3R antibody. Figure 3B shows four representative pictures of M3R and IgG staining on unfixed HSG cells taken at a ×400 magnification. As shown in Figure 3B, red signals indicate M3R staining on HSG cells (red arrows). Yellow colors indicate co-localization of IgG and M3R (b, c, d, e, and h, yellow arrows). Some staining showed M3R and IgG signal next to each other (a, f, and g, white arrows). In addition, M3Rs formed a cluster (b, c, e and h) with IgG. Interestingly, some of the IgG staining on the HSG cells showed lack of co-localization with M3R (green arrows). This result may suggest that SjS IgG binds to unidentified receptors or proteins on the surface of HSG cells, indicating an existence of other cell surface antigens in addition to M3R.

### Anti-M3R autoantibody positive SjS plasma inhibited carbachol-induced hAQP5 trafficking to the apical membrane

**Figure 4:**
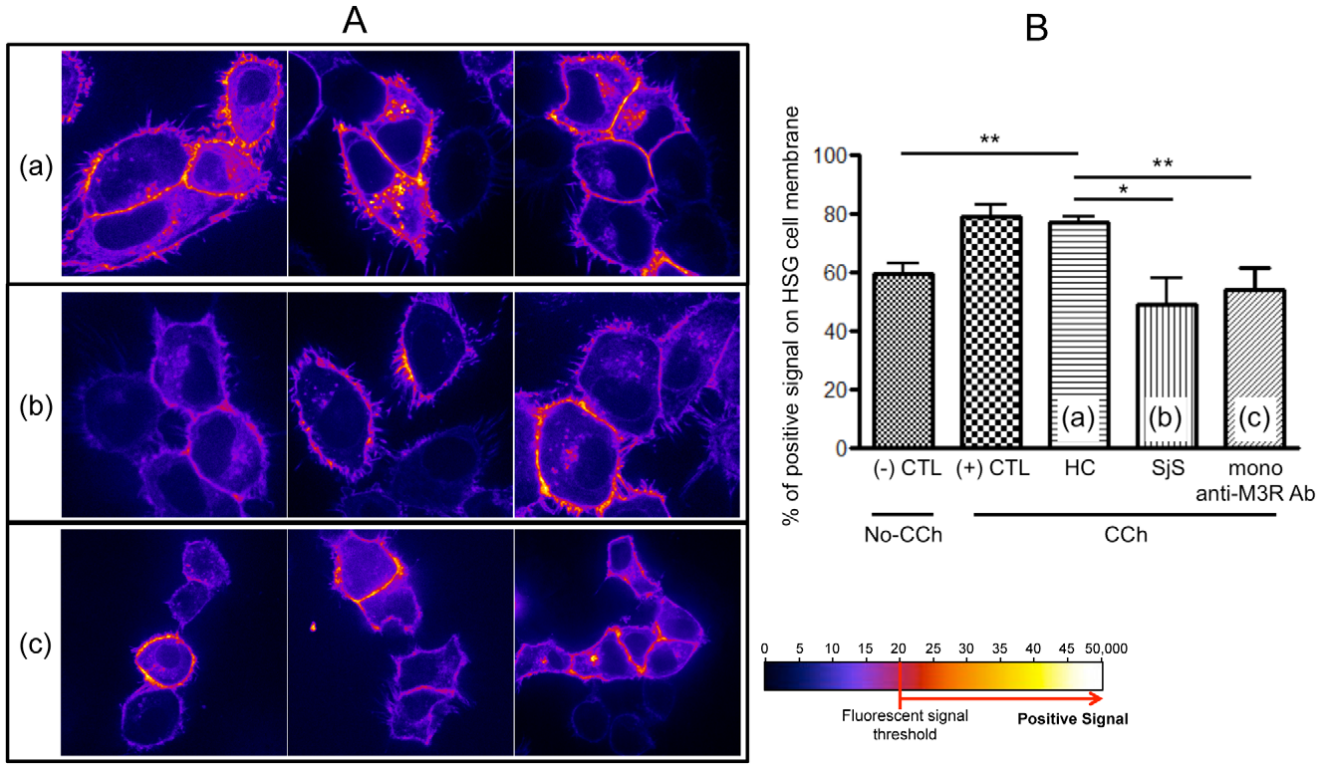
Inhibition of AQP5 trafficking to HSG cell plasma membrane by SjS plasma. (A) rhAQP5 vector transfected HSG cells were pre-incubated with five different healthy control (HC, (a)), five SjS plasma (b), or mono-clonal anti-M3R antibody (c) for 24 hours (only three representative pictures are shown). rhAQP5 GFP signal trafficking in HSG cells after CCh treatment were acquired with a spinning disk confocal microscope with a 63X oil-immersion objective, using a cascade-cooled EMCCD camera. HSG cells were observed at 30-second intervals for 15 minutes with 1/250 second fixed exposure time. Representative still images were captured after 10 minutes and rhAQP5 signals were converted and analyzed by ImageJ software (1.46a, open source software, http://rsb.info.nih.gov/ij/). Pictures were converted into ‘Fire’ 3D LUT (lookup tables) setting in ImageJ software and only GFP signal above the fluorescent signal threshold (≥80, red or yellow color in converted picture), was considered for quantitative analysis (% of positive signal on the membrane) in (B) to compare rhAQP5 trafficking among CCh treated groups. Each column represents the mean ± SEM of 5, p value less than 0.05 was considered significantly different by two-tailed unpaired Student's t-test, **p<0.01.

Following verification of M3R expression and CCh-induced rhAQP5 trafficking in HSG cells and the presence of anti-M3R autoantibodies in patient plasma samples, healthy controls (HC) and SjS plasma samples were screened for their ability to translocate hAQP5 to the cell membrane. Transfected HSG cells were pre-incubated with plasma from five HC or five SjS patients for 24 hours. hAQP5 trafficking in rhAQP5-HSG cells after CCh treatment was monitored by a confocal microscope (X630) for 10 minutes with 1/250 fixed exposure time. Images in Figure 4A show representative and converted images of AQP5 trafficking in HSG cells pre-incubated with HC, SjS plasma, or monoclonal anti-M3R antibody (Figure 4A-(a), (b), or (c), respectively) following CCh stimulation. Quantification of these results (Figure 4B) confirmed that CCh-induced hAQP5 trafficking was observed as indicated by increased signal from approximately 58 to 78% in cells incubated with no-plasma or HC plasma (p<0.01, Student's t-test). However, HSG cells pre-incubated with SjS plasma or monoclonal anti-M3R antibody showed reduced or maintained signals (50 or 54%, respectively), less hAQP5 trafficking, with no statistical difference compared with non-CCh treated rhAQP5-HSG (58%). These results indicate that autoantibodies in SjS patients' plasma, which can bind to M3R, inhibit hAQP5 trafficking from the cytoplasmic compartment to the plasma membrane in HSG cells following agonist stimulation.

### Anti-M3R autoantibody-positive SjS plasma inhibited carbachol-induced intracellular calcium release in HSG cells

**Figure 5:**
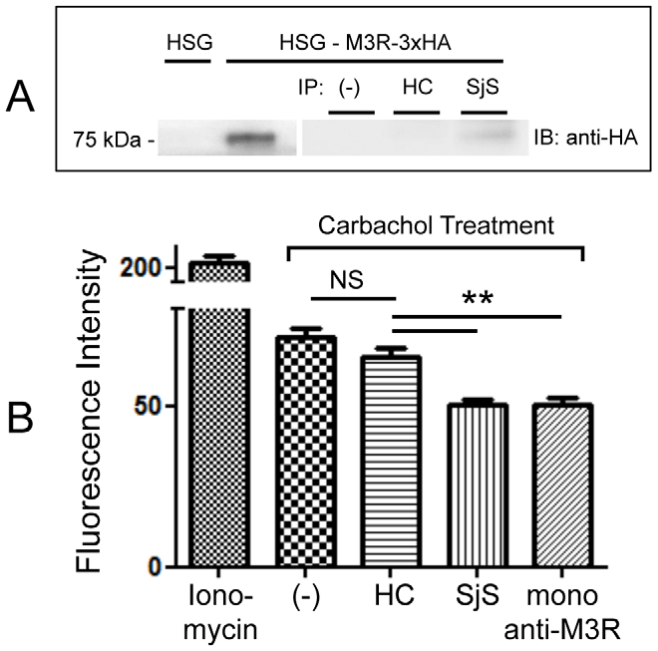
Reduced CCh-induced intracellular calcium releases by SjS IgG. (A) Immunoprecipitation (IP)-Western blot analysis using an extract of M3R-3xHA vector transfected HSG cells and pooled plasma samples from five HC or SjS plasma demonstrated that IgG in SjS plasma immunoprecipitated HA-tagged M3R (75 kDa), which was recognized by mouse monoclonal anti-HA antibodies. (B) HSG cells were pre-incubated with media only (negative control) or IgG from HC (10 μg/ml), SjS (10 μg/ml) or monoclonal anti M3R antibody (10 μg/ml) for 24 hours and treated with ionomycin (100 μM) or carbachol (100 μM). Data are based on three independent experiments (n = 5). Each column represents the mean ± SEM, p value less than 0.05 was considered significantly different by one-way ANOVA test.

To confirm the existence of autoantibodies that bind to M3R on HSG cells by immunoprecipitation, commercially available HA-tagged M3R expression vector was used to overexpress M3R in HSG cells. Figure 5A shows, immunoprecipitation (IP)-Western blot analysis using an extract of M3R-3xHA vector transfected HSG cells with pooled plasma samples from HC or SjS. HA-tagged M3R was detected by anti-HA antibody, showing a strong band at 75 kDa on western blot. More interestingly, only SjS plasma immunoprecipitated HA-tagged M3R, which was recognized by mouse monoclonal anti-HA antibody, compared to the transfected cells pre-incubated with HC plasma.

It has been known that the major signal transduction pathway though M3R activation by carbachol treatment leads to increased intracellular Ca^2+^ level. To test the impact of anti-M3R autoantibodies on intracellular Ca^2+^ level upon CCh treatment, rhAQP5-HSG cells were pre-incubated with media only (negative control) or purified IgG from HC (10 μg/ml), SjS (10 μg/ml), or monoclonal anti-M3R antibody (10 μg/ml) for 24 hours and stimulated with carbachol (100 μM). As a positive control, rhAQP5-HSG cells were incubated with ionomycin (100 μM) in the absence of any IgG. As shown in Figure 5B, intracellular calcium release in rhAQP5-HSG cells pre-incubated with IgG from SjS was significantly reduced in comparison with cells pre-incubated with HC IgG. As expected, this suppression by SjS IgG was also compatible with the cells pre-incubated with monoclonal anti-M3R antibody.

### SjS plasma inhibits CCh-induced cell volume decrease in AQP5-GFP transfected HSG cells

**Figure 6:**
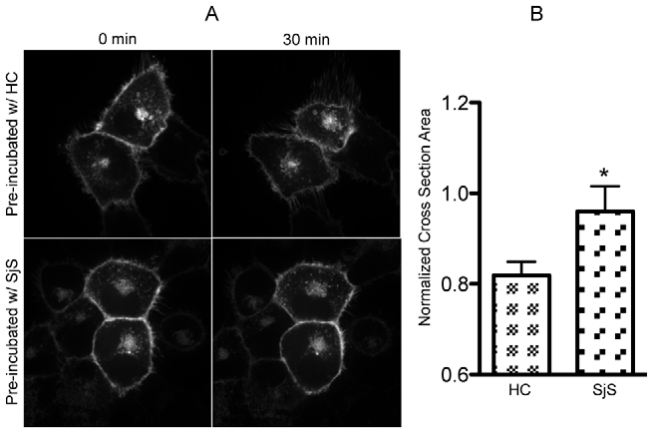
Inhibition of CCh-induced hAQP5-GFP transfected HSG cell shrinkage by SjS plasma. (A) hAQP5-GFP transfected HSG cells were pre-incubated with HC or SjS plasma for 24 hours and stimulated with CCh. Representative still images were captured before and after 30 minutes of CCh treatment. (B) Cell volume changes were quantified by measuring the areas within the cell membrane before and after CCh stimulation and normalized by the area before stimulation. Cross section of the cell at center of z-axis was considered for the analysis. Data are based on three independent experiments and all cells under a microscopic view were counted for calculation each time. Each column represents the mean ± SEM, p value less than 0.05 was considered significantly different by two-tailed unpaired Student's t-test, *p<0.05.

In the same experimental set-up as above in Figure 4, we observed unexpectedly that rhAQP5-HSG cells that were either pre-incubated with HC plasma or only with media exhibited decreased cell volume after CCh stimulation. However, SjS plasma pre-treated rhAQP5-HSG cells stimulated with CCh exhibited no significant cell volume change. The images from confocal microscope before and 30 minutes after CCh treatment on hAQP5-transfected HSG cells are presented in Figure 6A. For image quantitation, the area of cross-section at the center of Z-axis in the confocal image of the HSG cell was considered and measured before and after CCh stimulation. Cross sectioned area of each cells treated with CCh was normalized against the cross-sectioned area of cells before CCh stimulation (Figure 6B). rhAQP5-HSG cells pre-incubated with HC plasma decreased cell volume by approximately 20% (Real-time image in Movie S1) while transfected cells incubated with SjS showed negligible volume change.

## Discussion

Our previous studies and others' have shown that circulating autoantibodies specific for cell surface receptor M3R are one of the important factors in the pathogenesis of secretory dysfunction in SjS along with T- and B-cell infiltration in the exocrine glands [6], [29], [30]. The purpose of this study was to further define the roles of anti-M3R autoantibodies in SjS and their impact on AQP5 trafficking to the plasma membrane, which is critical for fluid secretion in the salivary glands. Although previously reported data showed discrepancies with respect to altered AQP5 trafficking in the SjS salivary glands, our data demonstrated 1) Acetylcholine receptor agonist CCh induced proper trafficking of human AQP5 in rhAQP5-vector transfected HSG cells; 2) Pre-incubation of M3R specific antagonist, 4-DAMP, prior to CCh stimulation inhibited AQP5 trafficking, implying AQP5 trafficking to the membrane is mainly mediated by M3R; 3) Anti-M3R autoantibodies and other cell surface antigen-specific antibodies are present in SjS plasma or IgG; 4) AQP5 trafficking to the membrane is suppressed when HSG cells were pre-incubated with SjS plasma or SjS IgG prior to CCh treatment; 5) CCh-induced volume reduction of AQP5 transfected cells are inhibited by SjS plasma.

It has been proven that M3R stimulation by neurotransmitters or CCh, non-selective agonist for MR, can increase intracellular Ca^2+^ level in salivary gland acinar cells and HSG cells [27], [31]. In addition, Nagy et al. confirmed 4-DAMP blocked increase of free intracellular Ca^2+^ level in CCh-treated HSG cells in a M3R-specific manner [27]. In rat parotid glands, AQP5 was translocated to the apical membrane with the treatment of epinephrine and cevimeline, which also increase the intracellular Ca^2+^ level [32], [33]. Furthermore, rat AQP5 cDNA transfected HSG cells were able to move AQP5 to the plasma membrane when triggered by increased intracellular Ca^2+^ [15]. These studies indicate increased intracellular Ca^2+^ level by agonist stimulation is essential in translocating AQP5 to the cell membrane. To verify the inhibitory effect of anti-M3R autoantibodies on AQP5 trafficking to the cell membrane of HSG cells, we stimulated the transfected cells with CCh to increase intracellular Ca^2+^ and monitored subsequent AQP5 trafficking in the presence or absence of SjS plasma under a confocal imaging system.

In this current study, we confirmed the presence of M3R by immunocytochemistry and semi-quantitative RT-PCR. M1R and M3R mRNA both are endogenously expressed in HSG cells with M3R more predominantly expressed. A monoclonal antibody specific for the second extracellular loop of M3R also identified M3R expression in our analyses. When the cell membrane fraction was purified from the M3R transfected cells, expression of AQP5 was increased in the membrane upon carbachol treatment, confirming the translocation of AQP5 to the cell surface. Blockage of this process by a M3R inhibitor but not by a M1R inhibitor clearly indicates that this trafficking is mainly mediated through M3R. Throughout the study, we have used our threshold signal value to define positive signal (A signal bar is provided in Figure where appropriate). In addition, we identified five SjS plasma samples that are positive for anti-M3R autoantibodies by co-immunostaining. The presence of IgG specific for cell surface antigens were undetected after pre-absorption or IgG depletion as indicted in our Figure 3. The specificity of these plasma samples was further verified with our immunoprecipitation assay with HA-tagged M3R and co-staining with monoclonal anti-M3R antibody. These results support our previous findings where we showed that anti-M3R antibodies in patients with primary Sjögren's syndrome desensitize M3R signal pathway, which confirmed by functional assays using carbachol-evoked responses of mouse-bladder smooth muscle strips [6]. Interestingly, our study identified cell surface antigen(s) other than M3R. To date, M3R is only cell surface antigen known in SjS while all others are intracellular antigens. Although identification of these cell surface antigens was not further pursued in our study for it was not a scope of this study, it would be important to characterize these cell surface antigen(s) due to their potential roles in SjS pathogenesis.

Altered expression of AQP5 in the salivary glands of SjS has been controversial. Immunofluorescence staining of AQP5 in acinar cells showed impaired distribution; reduced at the apical but increased at the basal membrane in SjS mouse (NOD) salivary glands [23], [34]. The treatment of purified SjS IgG to rat parotid acinar cells confirmed reduced trafficking of AQP5 into the apical membrane [5]. Abnormal distribution of AQP5 in minor salivary gland [20] or defective cellular trafficking in lacrimal glands [21] was also detected in SjS patients by immunohistochemical staining using anti-human AQP5 antibody. However, a study reported that there were no abnormal distributions of AQP5 detected in the salivary and lacrimal glands of SjS patients when anti-rat AQP5 antibody was used [22]. Thus, detection of AQP5 expression to the apical membrane appears to depend on the type of cell lines, analysis methods, and sensitivity and specificity of antibodies.

To measure the effect of SjS autoantibodies on AQP5 more directly, we monitored trafficking of AQP5 by expressing human AQP5 in a human cell line rather than measuring static expression of AQP5. This is the first report to our knowledge that demonstrates suppression of human AQP5 trafficking in the presence of SjS autoantibodies. In our analyses, AQP5 trafficking after CCh stimulation was greatly inhibited by pre-incubation with SjS plasma while HC plasma had no effect in this process. This effect was consistent with the cells pre-incubated with monoclonal anti-M3R antibody specific for the second extracellular loop of M3R in our experiment. This may imply that potential epitope(s) that antibodies in SjS plasma used in our experiment recognize may reside within the second extracellular loop of M3R as others have suggested [8], [28], [35], [36]. These results support our hypothesis that salivary gland dysfunction in SjS is mediated, in part, through the binding of anti-M3R autoantibodies to salivary gland acinar cells, resulting in inhibition of intracellular Ca2^+^ release and consequently AQP5 trafficking. It is presumed that since not all patients are positive for anti-M3R antibodies, the contribution of infiltrating cells in the glands to the loss of secretory function would be more significant in patients who are negative for autoantibodies whereas the roles of autoantibodies in dryness are more prominent in patients who are negative for focal infiltration. In addition, it is possible that patients who are positive for this antibody may have more significant correlation with certain clinical symptoms such as increased mild cognitive impairment or over-reactive bladder symptoms due to the autoantibodies targeting the receptors in the brain and the bladder, respectively. These possibilities are highly speculative at this point due to absence of specific and sensitive diagnostic assays but important to mention here to promote future studies. Roescher N et al. have shown that there are limitations to detect anti-M3R autoantibodies by using peptide-based ELISA system [37]. Thus, the development of reliable diagnostic tools for this antibody and further investigations on the subject is imperative and will warrant their associations with clinical symptoms.

One of the unexpected results was cell shrinkage observed with CCh stimulation of hAQP5-GFP transfected HSG cells. The transfected cells or the cells with HC plasma decreased cell volume by about 20% following CCh stimulation. Interestingly, this phenomenon was not observed in control GFP vector transfected HSG cells, which do not over-expressing AQP5 (data not shown). hAQP5-GFP transfected HSG cells slowly recovered the volume after CCh stimulation and no cells were identified as apoptotic during 45 minutes of monitoring, indicating cell shrinkage is not due to cell death. In vivo (or ex vivo) studies using single salivary gland acinar cell have shown that CCh induced intracellular Ca^2+^ release followed by cell shrinkage for the ion exchange and water efflux [38], [39]. However, HSG cells are known to be sensitive to osmolality changes in the suspending medium but are insensitive to cytoplasmic Ca^2+^ concentration [40], [41]. Taken together, AQP5 must be present in order for HSG cells to shrink in response to CCh stimulation and this process may not directly related to cytoplasmic Ca^2+^ concentration but is associated with changes in osmolality by CCh treatment. It has been reported that impaired cell volume regulation and trafficking to apical membrane were found in AQP5-deficient mice or cell lines [24], [42], [43]. Whether the findings with HSG cells presented here (i.e., SjS plasma or IgG suppresses CCh-induced cell volume change in AQP5-GFP transfected HSG cells) would directly lead to clinical implications is currently not known.

In conclusion, to understand one of the mechanisms of secretory dysfunction in autoimmune SjS, we monitored trafficking of human AQP5 in HSG cells following CCh stimulation in the presence or absence of SjS plasma and verified SjS IgG specific for M3R and other unidentified surface antigens. The binding of the autoantibodies to the receptor inhibited human AQP5 translocation to the plasma membrane. In addition, AQP5-overexpressed HSG cells decreased its volume following CCh treatment only in AQP5 transfected cells, which was also suppressed in the presence of SjS plasma. These alterations caused by anti-M3R autoantibodies in SjS plasma or IgG may play a critical role in secretory dysfunction in autoimmune SjS. Furthermore, our current study emphasizes the importance of future investigations on clinical associations between SjS symptoms, such as degree of secretory dysfunction, cognitive impairment, and/or bladder irritation, and different profiles (titers, isotypes, and/or specificity) of anti-M3R autoantibodies in individuals with SjS.

## Supporting Information

Figure S1 **Dominant expression of muscarinic type 3 receptor in human salivary gland** (**HSG**) **cell line.** MR expression was measured in HSG cells by semi-quantitative RT-PCR with M3R being the major subtype followed by M1R. M2R, M4R, and M5R were not detected in HSG cells. Reverse transcription polymerase chain reaction (RT-PCR) was performed using primers as follows: M1R: forward: 5′-TGGTGATCAAGATGCCAATGGTGG-3′, reverse: 5′-GAAGGCTTTGTTGCAGAGTGCGTA-3′; M2R: forward: 5′-CATATCCCGAGCCAGCAAGAGC-3′, reverse: 5′-GAGGCAACAGCACTGACTGAGG-3′; M3R: forward: 5′-CGAGACGAGAGCCATCTACTCC-3′, reverse: 5′-GACCAGGGACATCCTTTTCCGC-3′; M4R: forward: 5′-AGATTGTGACGAAGCAGACAGGCA, reverse: 5′-TTTAAAGGTGGCGTTGCACAGAGC-3′; M5R: 5′-GACCAACAATGGCTGTCACAAGGT-3′, reverse: 5′-TCTGTTGCAGAGGGCATAGCAGAT-3′. All primers used in this study were obtained from Integrated DNA Technologies. PCR conditions were 5 min at 95°C, (30 s at 95°C, 30 s at 57°C, and 30 s at 72°C) ×30 times, and 7 min at 72°C.
(TIF)

Figure S2 **Suppressed trafficking of rhAQP5 upon CCh stimulation in the presence of SjS plasma.** rhAQP5-vector transfected HSG cells were pre-incubated with HC (A) or SjS (B) plasma for 24 hours. (A) Strong yellow signal in the center of cells was reduced as the orange signal on the membrane was increased upon CCh stimulation of HC pre-incubated cells. (B) There was no difference in signal detected before and after CCh stimulation in the cytoplasm and the plasma membrane of SjS plasma incubated cells. rhAQP5 GFP signal in HSG cells after CCh treatment was acquired with a spinning disk confocal microscope with a 63X oil-immersion objective, using a cascade-cooled EMCCD camera. HSG cells were observed at 30-second intervals for 30 minutes with 1/250 second fixed exposure time. Representative still images were captured after 0 and 30 minutes and rhAQP5 signals were converted and analyzed by ImageJ software (1.46a, open source software, http://rsb.info.nih.gov/ij/). Pictures were converted into ‘Fire’ 3D LUT (lookup tables) setting and signal spreading in a cell was measured with interactive 3D surface plot v2.3.3 in ImageJ software.
(TIF)

Movie S1 **Increased expression of rhAQP5 on HSG cell membrane and cell volume change following CCh stimulation.** (A) Live image of increased AQP5 expression on the membrane of HSG cells and its shrinkage upon CCh treatment. (B) Converted image of (A). (C) Live image of transfected HSG cells pre-incubated with HC. (D) Live image of transfected HSG cells pre-incubated with SjS plasma. Original live images were converted into ‘Fire’ 3D LUT (lookup tables) setting in ImageJ software.
(MOV)
