# Supplementary figures and images for: Autoantibodies against Muscarinic Type 3 Receptor in Sjögren's Syndrome Inhibit Aquaporin 5 Trafficking
Source: PLoS One. 2013 Jan 30;8(1):e53113. doi: 10.1371/journal.pone.0053113 (PMC3559734; doi:10.1371/journal.pone.0053113)

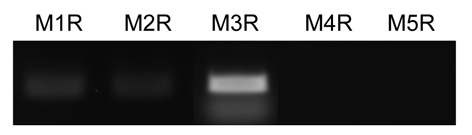

Supplement: Figure S1 — Dominant expression of muscarinic type 3 receptor in human salivary gland (HSG) cell line. MR expression was measured in HSG cells by semi-quantitative RT-PCR with M3R being the major subtype followed by M1R. M2R, M4R, and M5R were not detected in HSG cells. Reverse transcription polymerase chain reaction (RT-PCR) was performed using primers as follows: M1R: forward: 5′-TGGTGATCAAGATGCCAATGGTGG-3′, reverse: 5′-GAAGGCTTTGTTGCAGAGTGCGTA-3′; M2R: forward: 5′-CATATCCCGAGCCAGCAAGAGC-3′, reverse: 5′-GAGGCAACAGCACTGACTGAGG-3′; M3R: forward: 5′-CGAGACGAGAGCCATCTACTCC-3′, reverse: 5′-GACCAGGGACATCCTTTTCCGC-3′; M4R: forward: 5′-AGATTGTGACGAAGCAGACAGGCA, reverse: 5′-TTTAAAGGTGGCGTTGCACAGAGC-3′; M5R: 5′-GACCAACAATGGCTGTCACAAGGT-3′, reverse: 5′-TCTGTTGCAGAGGGCATAGCAGAT-3′. All primers used in this study were obtained from Integrated DNA Technologies. PCR conditions were 5 min at 95°C, (30 s at 95°C, 30 s at 57°C, and 30 s at 72°C) ×30 times, and 7 min at 72°C. (TIF) [file pone.0053113.s001.tif]

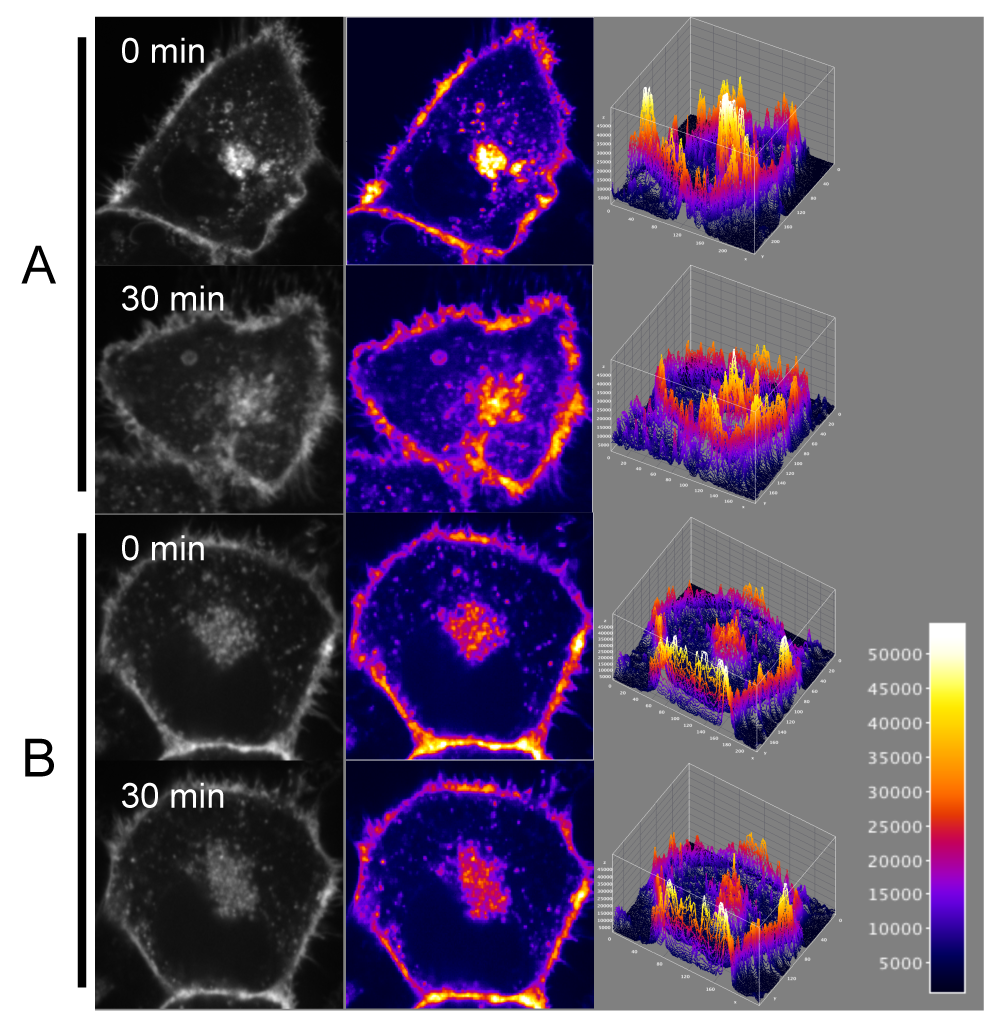

Supplement: Figure S2 — Suppressed trafficking of rhAQP5 upon CCh stimulation in the presence of SjS plasma. rhAQP5-vector transfected HSG cells were pre-incubated with HC (A) or SjS (B) plasma for 24 hours. (A) Strong yellow signal in the center of cells was reduced as the orange signal on the membrane was increased upon CCh stimulation of HC pre-incubated cells. (B) There was no difference in signal detected before and after CCh stimulation in the cytoplasm and the plasma membrane of SjS plasma incubated cells. rhAQP5 GFP signal in HSG cells after CCh treatment was acquired with a spinning disk confocal microscope with a 63X oil-immersion objective, using a cascade-cooled EMCCD camera. HSG cells were observed at 30-second intervals for 30 minutes with 1/250 second fixed exposure time. Representative still images were captured after 0 and 30 minutes and rhAQP5 signals were converted and analyzed by ImageJ software (1.46a, open source software, http://rsb.info.nih.gov/ij/). Pictures were converted into ‘Fire’ 3D LUT (lookup tables) setting and signal spreading in a cell was measured with interactive 3D surface plot v2.3.3 in ImageJ software. (TIF) [file pone.0053113.s002.tif]
